# Supplementary material for: Classification of the trajectory of changes in food intake in special nursing home for oldest-old in the 6 months before death: A secondary analysis
Source: PLoS One. 2025 Apr 9;20(4):e0319669. doi: 10.1371/journal.pone.0319669 (PMC11981222; doi:10.1371/journal.pone.0319669)

Appendix 1. The clusters appeared by the analysis with the number of clusters from 2 to 6

Cluster 2

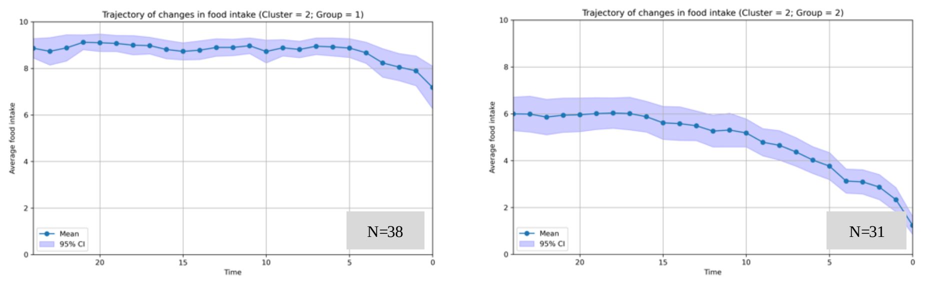

Cluster 3

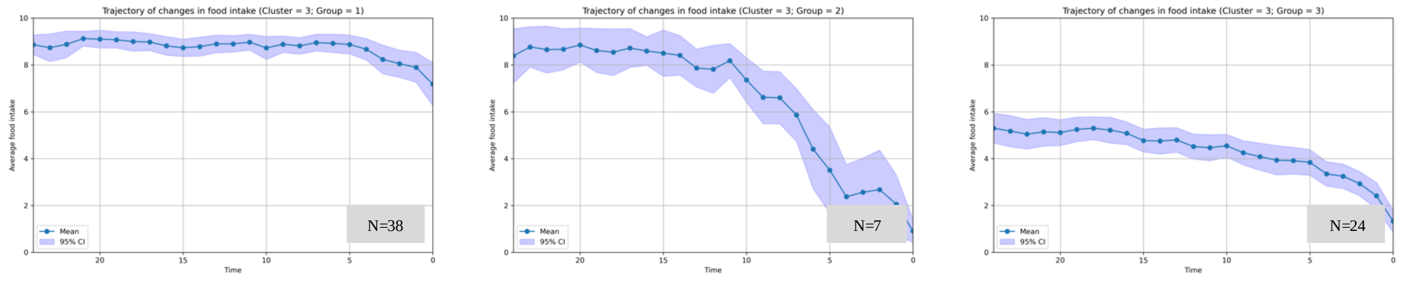

Cluster4

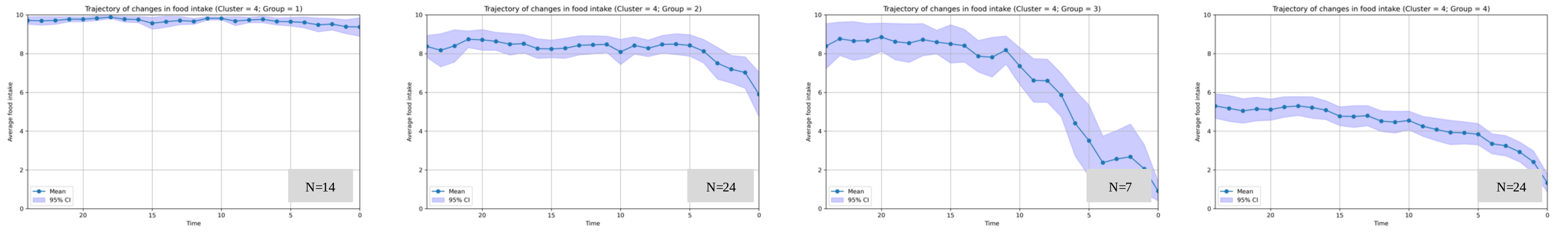

Cluster5

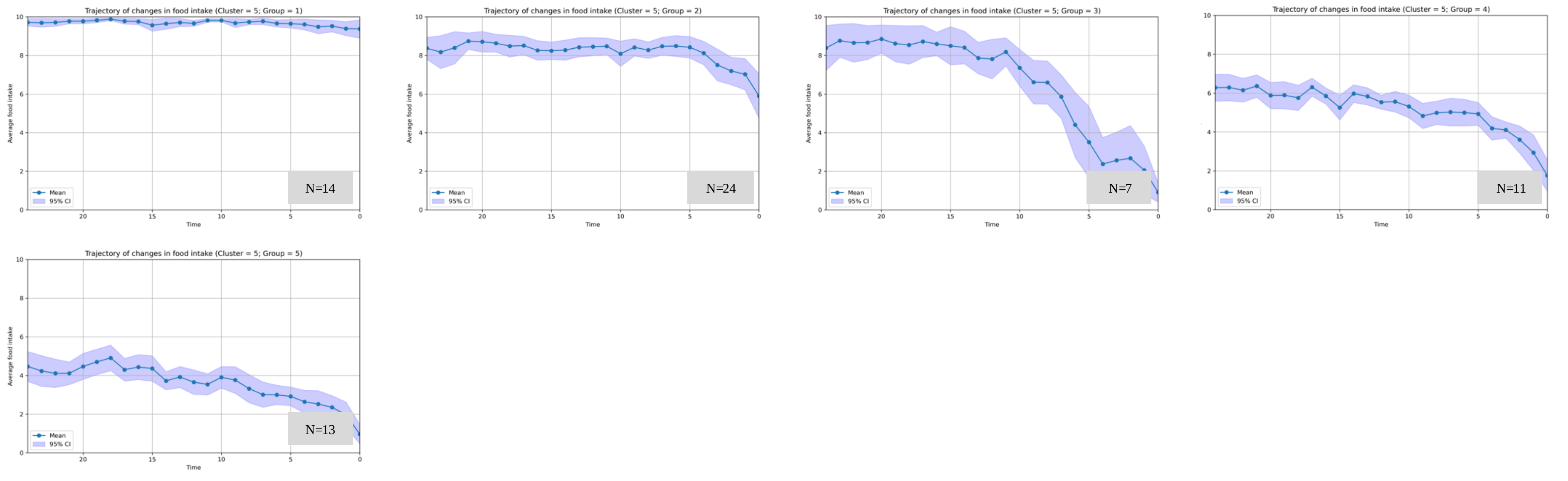

Cluster6

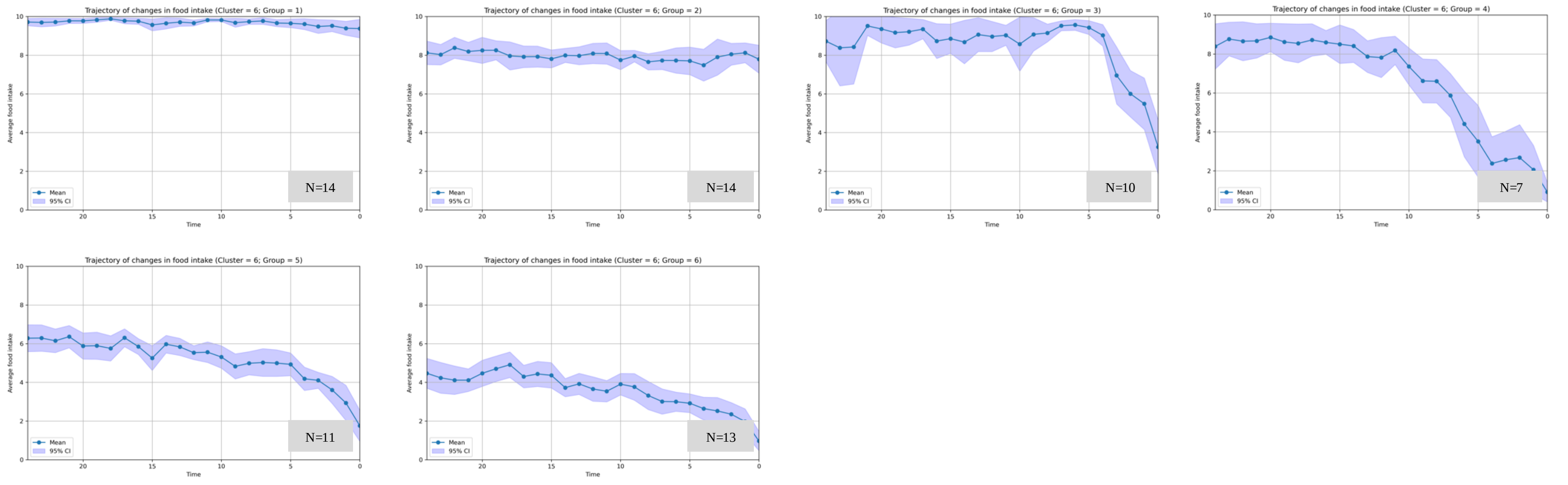

Supplement: S1 Appendix — (PDF) [file pone.0319669.s001.pdf]
